# Immunogenicity of prostate cancer is augmented by BET bromodomain inhibition

**DOI:** 10.1186/s40425-019-0758-y

**Published:** 2019-10-25

**Authors:** Wendy Mao, Ali Ghasemzadeh, Zachary T. Freeman, Aleksandar Obradovic, Matthew G. Chaimowitz, Thomas R. Nirschl, Emily McKiernan, Srinivasan Yegnasubramanian, Charles G. Drake

**Affiliations:** 1 Department of Oncology, Sidney Kimmel Comprehensive Cancer Center, Johns Hopkins University School of Medicine, Baltimore, MD 21287 USA; 2 Columbia Center for Translational Immunology, Columbia University Medical Center, New York, NY 10032 USA; 3 Unit for Laboratory Animal Medicine (ULAM), Michigan Medicine, University of Michigan, Ann Arbor, MI 48109 USA; 4 Rogel Cancer Center, Michigan Medicine, University of Michigan, Ann Arbor, MI 48109 USA; 5 Columbia University Systems Biology, Herbert Irving Cancer Research Center, Columbia University Medical Center, New York, NY 10032 USA; 6 Department of Urology, Columbia University Medical Center, New York, NY 10032 USA; 7 Herbert Irving Comprehensive Cancer Center, Division of Hematology / Oncology, Columbia University Medical Center, 177 Fort Washington Avenue, Suite 6GN-435, New York, NY 10032 USA

**Keywords:** Epigenetic, BET, Bromodomain, Cancer immunology, Immunotherapy, Immune checkpoint blockade, Prostate cancer, CTLA-4, Treg, Immune checkpoint inhibition

## Abstract

**Background:**

Prostate cancer responds poorly to current immunotherapies. Epigenetic therapies such as BET Bromodomain inhibition can change the transcriptome of tumor cells, possibly making them more immunogenic and thus susceptible to immune targeting.

**Methods:**

We characterized the effects of BET bromodomain inhibition using JQ1 on PD-L1 and HLA-ABC expression in two human prostate cell lines, DU145 and PC3. RNA-Seq was performed to assess changes on a genome-wide level. A cytotoxic T cell killing assay was performed in MC38-OVA cells treated with JQ1 to demonstrate increased immunogenicity. In vivo experiments in the Myc-Cap model were conducted to show the effects of JQ1 administration in concert with anti-CTLA-4 checkpoint blockade.

**Results:**

Here, we show that targeting BET bromodomains using the small molecule inhibitor JQ1 decreased PD-L1 expression and mitigated tumor progression in prostate cancer models. Mechanistically, BET bromodomain inhibition increased MHC I expression and increased the immunogenicity of tumor cells. Transcriptional profiling showed that BET bromodomain inhibition regulates distinct networks of antigen processing and immune checkpoint molecules. In murine models, treatment with JQ1 was additive with anti-CTLA-4 immunotherapy, resulting in an increased CD8/Treg ratio.

**Conclusions:**

BET Bromodomain inhibition can mediate changes in expression at a genome wide level in prostate cancer cells, resulting in an increased susceptibility to CD8 T cell targeting. These data suggest that combining BET bromodomain inhibition with immune checkpoint blockade may have clinical activity in prostate cancer patients.

## Introduction

Prostate cancer is the second leading cause of cancer-related death in U.S. men [1]. Despite advances in immunotherapy, immune checkpoint blockade has yet to confer significant benefit for patients with prostate cancer [2–4]. This resistance to immunotherapy may be due in part to the fact that prostate cancer is poorly infiltrated by cytotoxic T-cells as compared to other solid tumors [4]. Since PD-1 / PD-L1 blockade functions by blocking the inhibitory interactions between tumor cells and T-cells, much of its mechanism of action relies on the presence of a pre-existing anti-tumor T cell response [5]. The lack of CD8 infiltration in prostate cancer may be attributable to several factors, including the presence of suppressive regulatory T-cells (Tregs) [6], and/or myeloid-derived suppressor cells (MDSCs) that serve to dampen cytotoxic effects [7]. Additionally, prostate tumor cells themselves commonly downregulate MHC Class I expression [8] to evade immune detection.

Recently, epigenetic modulating drugs have emerged as potential agents to reprogram tumor cells, reversing the acquired epigenetic changes that cancer cells accumulate throughout their evolution and progression [9, 10], including those that contribute to immune evasion. Of particular interest are epigenetic modifiers targeting bromodomains, protein domains that recognize acetylated lysine residues such as those on histone proteins which couple the “reading” of activating marks to the transcription machinery [11, 12]. The inhibition of BRD4, a member of the Bromodomain and ExtraTerminal (BET) family of bromodomain-containing proteins, was recently shown to reduce levels of Androgen Receptor (AR) driven target genes in prostate cancer, and reduce tumor burden in murine models [12]. Due to its role as a transcriptional regulator, it is possible that BRD4 recruitment mediates not only oncogenic drivers like AR [13] and MYC [14], but also plays a role in regulating immune networks as well.

To understand the effects of BET Bromodomain inhibition on the immunogenicity of prostate cancer, we performed a series of studies examining the effects of inhibition on the levels of immunologically relevant molecules. In addition, we tested whether BRD4 inhibition rendered tumor cells more susceptible to CD8-mediated lysis, both in vitro and in vivo.

## Results

### BET Bromodomain inhibition reduces PD-L1 expression

**Fig. 1:**
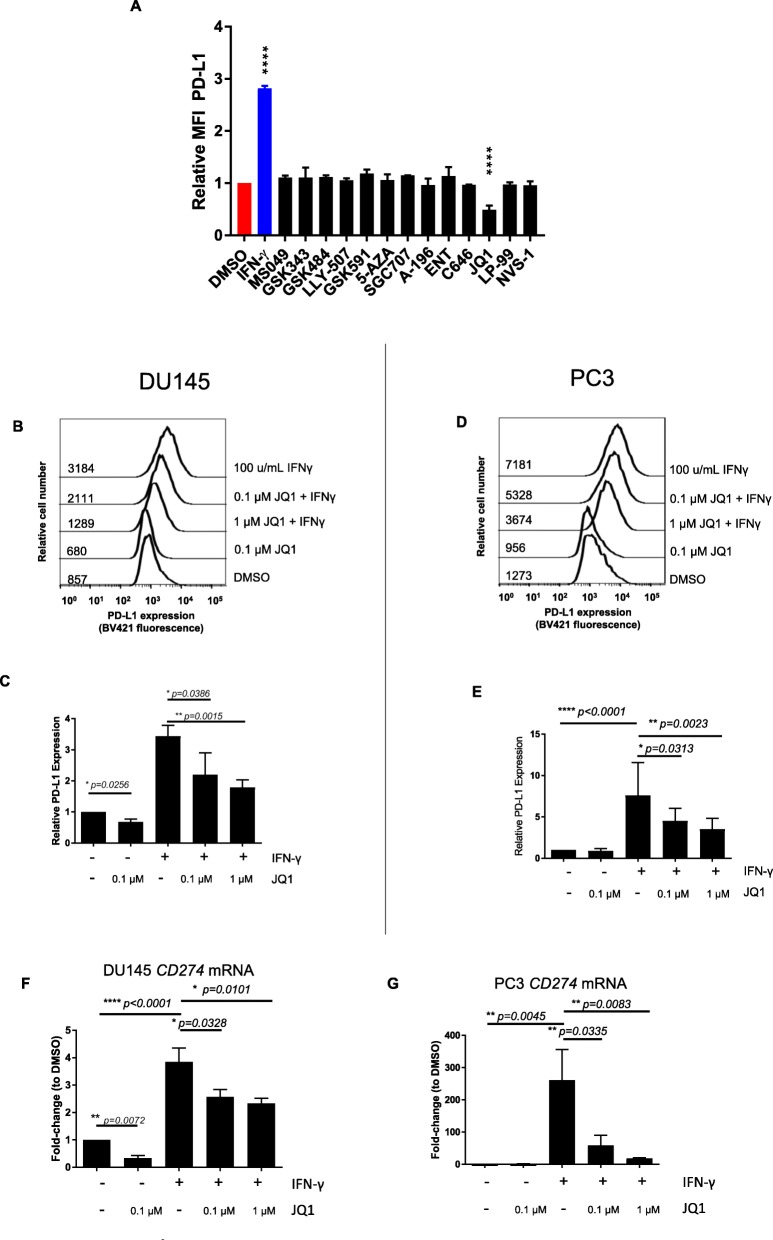
BET Bromodomain Inhibition Downregulates PD-L1 Expression in Prostate Cancer. **a** Relative PD-L1 MFI (normalized to DMSO control) in DU145 cells treated with indicated agents, gated on live cells. *N* = 1 sample / iteration, repeated × 3. **b** Representative histograms of PD-L1 expression in DU145 cells incubated with JQ1 and/or IFNγ gated on live cells. **c** Summary flow cytometry data for B, *N* = 1 sample / iteration, repeated × 8. **d** PD-L1 expression in PC3 cells incubated with JQ1 and/or IFNγ, gated on live cells. **e** Summary flow cytometry data for D, *N* = 1 sample / iteration, repeated × 8. **f** PD-L1 (CD274) mRNA levels in DU145. Fold-change normalized to 18 s and to control (DMSO) treatment. *N* = 3 / group, repeated × 2. **g** PD-L1 (CD274) mRNA levels in PC3. Normalized to control (DMSO). *N* = 3 / group, repeated × 2. * *p* < 0.05, **p < 0.01, *** *p* < 0.001, ****p < 0.0001. Error bars = standard deviation

To investigate the effects of epigenetic drugs on the immunogenicity of prostate cancer, we tested a panel of twelve different small-molecule inhibitors – these agents were selected to target a variety of epigenetic mechanisms including DNA methylation, histone deacetylation, and bromodomains (Additional file 1: Table S1). The primary readout for these studies was PD-L1 expression, since the PD-1 / PD-L1 interaction has emerged as a major immunosuppressive mechanism in several tumor types [15–17]. To model advanced prostate cancer, we used the human prostate cancer cell lines DU145 and PC3, both of which are negative for androgen receptor signaling [18]. As shown in Fig. 1a, the BET bromodomain BRD4 inhibitor JQ1 was the only small molecule inhibitor tested that suppressed PD-L1 expression significantly. We then chose two doses of JQ1, 0.1 μM and 1 μM for further testing based the ability of these dose levels to down-regulate PD-L1 with relatively minimal effects on proliferation (Additional file 2: Figure S1). Since PD-L1 expression in tumors is likely driven by IFN-γ in the tumor microenvironment (TME) [19, 20], we next tested whether bromodomain inhibition could inhibit IFN-γ mediated PD-L1 up-regulation. This was indeed the case, as JQ1 suppressed IFN-γ driven PD-L1 expression in both PC3 and DU145 cells in a dose-dependent manner (Fig. 1b-e). To support the specificity of this effect, we utilized a second small molecule inhibitor (RVX208); this agent inhibits the second bromodomain (BD2) of BET proteins with 170-fold more specificity than the first bromodomain (BD1) in targeted proteins [21]. RVX208 was comparable in activity to JQ1 in its ability to suppress PD-L1 expression in PC3 cells (Additional file 3: Figure S2A-B). Since BET bromodomains act at the transcriptional level [22], we quantified mRNA levels of CD274 (PD-L1) after drug exposure, and found that JQ1 reduced mRNA levels of CD274 in a dose-dependent manner (Fig. 1f-g). Taken together, these data show that BET bromodomain inhibition decreases PD-L1 expression in prostate cancer cells at both the protein and message level.

### BET Bromodomain inhibition increases MHC I expression

**Fig. 2:**
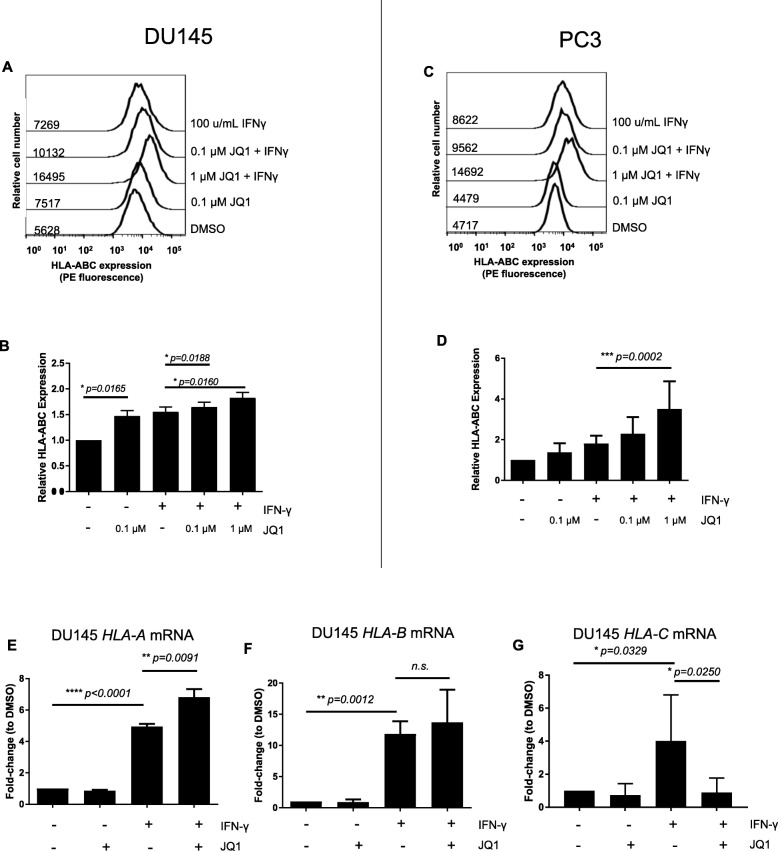
BET Bromodomain Inhibition increases MHC I Expression in Prostate Cancer. **a** Representative histograms of HLA-ABC expression on DU145 cells treated with JQ1 and/or IFNγ gated on live cells. **b** Summary flow cytometry data for A (normalized to DMSO control). *n* = 1 sample / iteration, repeated × 8. **c** Representative histograms of HLA-ABC expression in PC3 cells incubated with JQ1 and/or IFNγ gated on live cells. **d** Summary flow cytometry data for C (normalized to DMSO control). *n* = 1 sample / iteration, repeated × 8. **e** *HLA-A* mRNA levels in DU145. Fold-change normalized to 18 s and then normalized to control (DMSO) treatment. *n* = 3 / group, repeated × 2. **f** *HLA-B* mRNA levels in DU145. Fold-change normalized to 18 s and then normalized to control (DMSO) treatment. *n* = 3 / group, repeated × 2. **g** *HLA-C* mRNA levels in DU145 fold-change normalized to 18 s and then normalized to control (DMSO) treatment. *n* = 3 / group, repeated × 2. * *p* < 0.05, **p < 0.01, *** *p* < 0.001, ****p < 0.0001. Error bars = standard deviation

In addition to PD-L1, a number of immune molecules are important in CD8 T cell mediated anti-tumor effects. In particular, Class I MHC is required for CD8 T cell antigen recognition and like PD-L1, MHC expression is up-regulated by pro-inflammatory IFN-γ signaling [23]. If BET Bromodomain inhibition resulted in a global down-regulation of immune-related molecules (such as Class I MHC), it could be that the anti-tumor effects mediated by PD-L1 down-regulation would be attenuated by down-regulation of MHC. This was not the case - in the presence of IFN-γ, JQ1 exposure resulted in a further increase in Class I MHC expression (Fig. 2a-d). As the antibody used in these studies recognizes all 3 human HLA alleles of Class I, we next used qPCR to determine which specific alleles were involved. Interestingly, BET Bromodomain inhibition preferentially increased HLA-A and possibly HLA-B expression, while simultaneously mediating a decrease in HLA-C expression (Fig. 2e-g). These data suggest that the immune effects of BET bromodomain inhibition may be complex but highlight increased CD8 T cell recognition as one possible outcome.

### BET Bromodomain inhibition augments susceptibility to CD8 T cell-mediated cytotoxicity

**Fig. 3:**
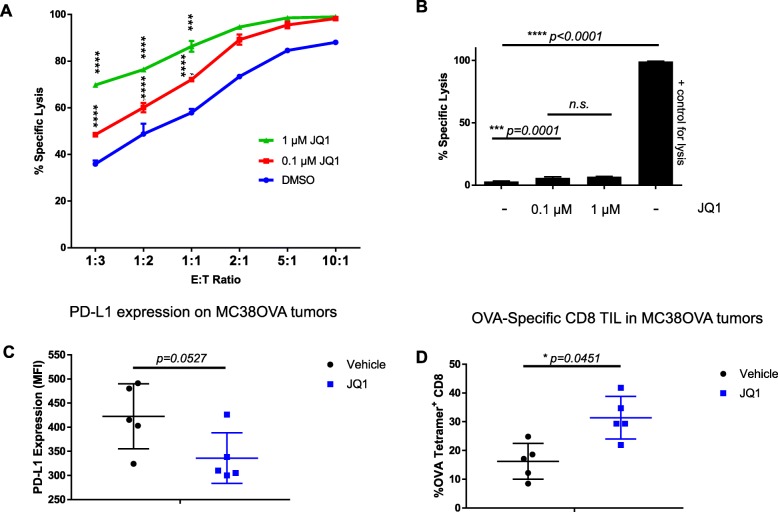
BET Bromodomain Inhibition Augments Susceptibility to CD8 T Cell-Mediated Cytotoxicity. **a** Specific lysis of JQ1 pretreated MC38OVA cells by OT-I CD8 cells. Specific lysis calculated as (% Lysis - %Nonspecific Lysis) / (% Maximum lysis - % nonspecific lysis). Significance compared to DMSO group. *N* = 3 wells / iteration, repeated × 3. **b** Percent specific lysis of pretreated MC38OVA cells in the absence of OT-I CD8 cells. Significance calculated using Tukey’s multiple comparisons test through 2-way Repeated Measures ANOVA . * *p* < 0.05, **p < 0.01, *** *p* < 0.001, ****p < 0.0001. Error bars = standard deviation. **c** Summary of flow cytometry data for PD-L1 staining in ex-vivo MC38OVA tumors, gated on Live CD45^−^ cells. *N* = 5 mice / iteration, repeated × 3. **d** Summary of flow cytometry data for OVA Tetramer staining in ex-vivo MC38OVA tumors gated on Live CD45^+^TCRb^+^CD4^−^CD8^+^ cells. *N* = 5 mice / iteration, repeated × 3

To test whether PD-L1 down-regulation and MHC Class I up-regulation potentiates CD8 T-cell mediated lysis, we employed an antigen-specific model system. For these studies, we focused on MC38-OVA, a murine colon cancer line that expresses the OVA peptide as a model antigen – potentially mimicking a mutation associated neoantigen (MANA) [24]. These cells express moderate PD-L1 levels at baseline, expression is augmented by IFN-γ, and like the human prostate cancer cells above, JQ1 was able to significantly decrease PD-L1 expression and increase expression of murine Class I (Additional file 4: Figure S3 A-D). For CD8 killing, we used transgenic OT-1 T cells, these cells recognize the OVA peptide SIINFEKL in the context of H-2K^b^ [25]. As expected, OT-1 T cells lysed OVA-expressing target cells, with increased killing at increased effector to target ratios (Fig. 3a). Treating target cells with JQ1 increased CD8-mediated tumor cell lysis; this was more pronounced at increased doses. To rule out the possibility that JQ1 itself mediates MC38 tumor cell lysis, we repeated these studies in the absence of antigen-specific CD8 T cells; as shown in Fig. 3b JQ1 treatment alone mediated only a moderate, dose-independent increase in baseline lysis. To test whether JQ1 treatment also results in increased immunogenicity in vivo, we treated mice with implanted MC38OVA. Tumors harvested from JQ1 treated mice showed a trend towards decreased PD-L1 expression (Fig. 3c). Additionally, in vivo treatment with JQ1 led to an increase of OVA-specific CD8 T cells (Fig. 3d) as assessed by OVA tetramer staining.

### BET Bromodomain inhibition modulates distinct networks of immune-related genes

**Fig. 4:**
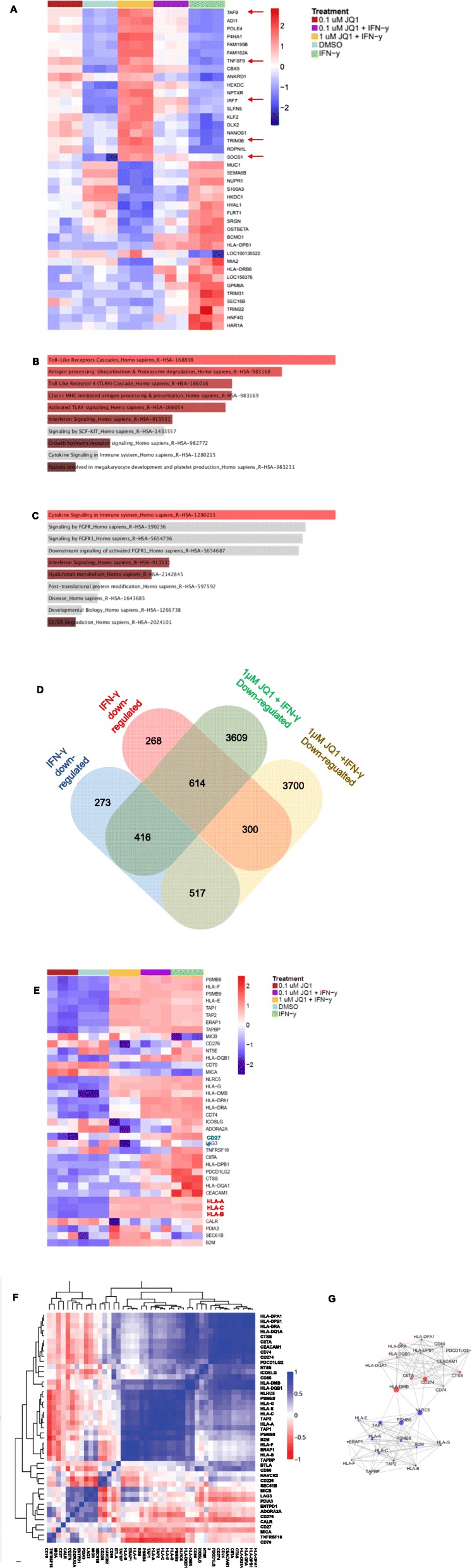
BET Bromodomain Inhibition Modulates Distinct Networks of Immune-Related Genes. **a** Top 20 upregulated and downregulated transcripts significantly altered by IFNγ significantly further modulated in the presence of 1uM JQ1 in DU145 cells. Scale represents log transformed FPKMs + 0.1, data are row normalized. **b** Gene set enrichment analysis (GSEA) using the Reactome database in EnrichR for top 20 genes whose expression is significantly altered by IFNγ and whose expression is upregulated by incubation with 1uM JQ1 in DU145 cells. Colors represent ranking based on combined enrichment score (a value calculated through multiplying log *p*-value and z-score), with highest enrichment scores in brighter shades. **c** GSEA using the Reactome database in EnrichR for top 20 genes whose expression is significantly altered by IFNγ and whose expression is downregulated by addition of 1uM JQ1 in DU145 cells. Colors represent ranking based on combined enrichment score, with highest enrichment scores in brighter shades. **d** Genes in DU145 cells significantly downregulated or upregulated with IFN-γ treatment whose expression is further modulated with JQ1 treatment. **e** Expression of immune-related genes, including Class I, Class II, and checkpoints, in DU145 cells across treatment conditions. Scale represents log transformed FPKMs + 0.1, data are row normalized. **f** Correlation matrix of immune-related genes in DU145 cells across treatment conditions. **g** Networks of immune-related genes, including Class I, Class II, and checkpoints, altered by JQ1 treatment. Lines indicate strength of connections between two genes, and size of node represents relative importance of gene in the network.*p* < 0.05 for all significantly differentially expressed transcripts. *N* = 3/group, repeated × 1

Given the observed increase in MHC Class I and decrease in PD-L1 expression, we hypothesized that BRD4 inhibition might modulate expression of additional immunologically related transcripts. To gain a better understanding of these effects on a genome-wide level, we used RNA sequencing to profile DU145 cells treated with JQ1 and IFN-γ. We focused downstream analyses on genes whose expression was modulated by IFN-γ treatment and sought to determine which of the IFN-γ modulated transcripts were further modulated by BET bromodomain inhibition. These data are shown in Fig. 4a; as one example, IRF7, a transcription factor which is responsible for transcription of Interferon Stimulated Genes (ISGs) [26], was slightly upregulated by IFN-γ, with expression further increased in a dose-dependent manner through BET bromodomain inhibition. Other immune genes further upregulated by BET bromodomain inhibition over IFN-γ include TNFSF9 (4-1BBL) and TRIM36, suggesting increased cytotoxic costimulatory ligand expression [27] and potentially improved Class I antigen processing [28]. SOCS1, a negative regulator of IFN-γ signaling [29], was also upregulated by BET bromodomain inhibition, possibly as a response to the increase in proinflammatory gene expression (Fig. 4a).

Gene set enrichment analysis (GSEA) on the top 20 upregulated and downregulated genes in the subset above (IFN-γ modulated transcripts that are further modulated by BET bromodomain inhibition) using the Reactome database [30, 31] revealed that the top 20 most upregulated genes were enriched for antigen presentation and TLR signaling pathways (Fig. 4b), whereas the top 20 most downregulated genes were associated with cytokine and growth factor signaling pathways (Fig. 4c). Of the 2388 genes whose expression was modulated by IFN-γ, BET Bromodomain inhibition further altered the expression of 1847 genes within that subset (Fig. 4d), confirming the broad immunological target spectrum of BET bromodomain inhibition. We next interrogated a selected set of immune-related genes commonly implicated in the anti-tumor response as well as antigen processing and presentation [32]. Consistent with the data in Fig. 2, these data confirmed that MHC Class I HLA-A and HLA-B transcripts were up-regulated by BET bromodomain inhibition; interestingly the opposite trend was noted for Class II (HLA-DPA1, HLA-DR) expression (Fig. 4e). BET bromodomain inhibition also down-modulated expression of the A2A receptor (ADORA2A); this receptor and others in the extracellular adenosine pathway may play a role in maintaining a suppressive tumor microenvironment [33]. We next performed correlation analysis to determine which immune-relevant transcripts were coordinately regulated. As shown in Fig. 4f, two distinct clusters of immune genes were observed, with one containing HLA and other genes related to antigen processing and presentation, and a second containing suppressive molecules like ADORA2A and ENTPD1 (CD39) (Fig. 4f). Network analysis of these data (Fig. 4g) showed that *CD274* (PD-L1) and *PDCD1LG2* (PD-L2) clustered with Class II genes, whereas Class I genes formed a separate node (Fig. 4g). Taken together, these data confirm the concept that BET bromodomain inhibition modulates the expression of a number of immunologically relevant transcripts and highlights the complexity of this regulation in that neither monotonic up-regulation nor down-regulation was observed.

### BET Bromodomain inhibition augments antitumor immunity and increases tumor infiltration

**Fig. 5:**
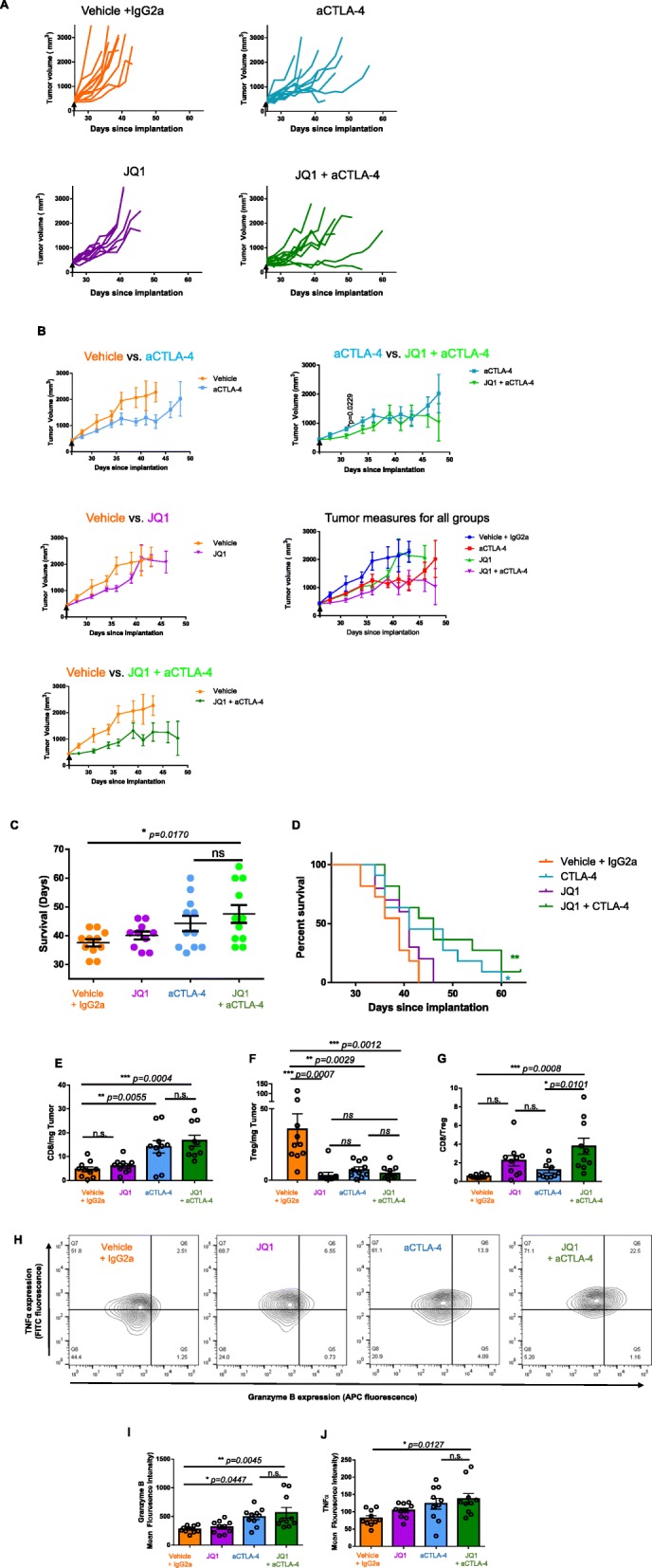
BET Bromodomain Inhibition Augments Antitumor Immunity and Increases Tumor Infiltration. **a** Volume of MycCap tumors treated as indicated, rx initiated d28 post-implatation. Each line represents an individual tumor. Arrows indicate start of treatment on d26. *N* = 10/group, repeated × 2. **b** Summary data of mean tumor growth for A. *N* = 10/group, repeated × 2. Error bars represent S.E.M. Arrows indicate start of treatment on d26. **c** Mean survival of animals with MycCap tumors in indicated treatment groups. *N* = 10/ group, repeated × 2. Lines indicate mean survival. Error bars represent S.E.M. **d** Survival of animals with MycCap tumors in indicated treatment groups. *N* = 10/group, repeated × 2. Lines indicate percent survival. Significance calculated using Log-rank (Mantel-Cox) test. **p = 0.0046 for Vehicle vs. JQ1 + aCTLA-4, *p = 0.0278 for Vehicle vs. aCTLA-4. No other comparisons between growth curves were significant. **e** Live CD45^+^TCRb^+^CD4^−^CD8^+^ (CD8) cells /mg tumor in MycCap tumors from animals treated as indicated. *N* = 10/group, repeated × 2. **f** Live CD45^+^TCRb^+^CD4^+^CD8^−^FoxP3^+^ (Treg) / mg tumor in MycCap tumors from animals in indicated treatment groups. *N* = 10/group, repeated × 2. **g** Ratio of live CD45^+^TCRb^+^CD4^−^CD8^+^ (CD8) cells to CD45^+^TCRb^+^CD4^+^CD8^−^FoxP3^+^ (Treg) cells in MycCap tumors from treatment groups indicated. *N* = 10/group, repeated × 2. **h** TNFα and Granzyme B secretion from stimulated live CD45^+^TCRb^+^CD4^−^CD8^+^ (CD8) cells in MycCap tumors from mice in treatment groups as indicated. Gates were set based on non-stimulated CD8 controls. **i** Summary of mean fluorescence intensity (MFI) of Granzyme B staining in stimulated live CD45^+^TCRb^+^CD4^−^CD8^+^ (CD8) cells in MycCap tumors from mice in treatment groups as indicated. *N* = 10 mice/group, repeated × 2. **j** Summary of mean fluorescence intensity (MFI) of TNFα staining in stimulated live CD45^+^TCRb^+^CD4^−^CD8^+^ (CD8) cells in MycCap tumors from mice in treatment groups as indicated. *N* = 10 mice/group, repeated × 2. * *p* < 0.05, **p < 0.01, *** *p* < 0.001, ****p < 0.0001. *P* values were calculated through one-way ANOVA. Error bars shown represent S.E.M

To quantify the in vivo effects of BET bromodomain inhibition on prostate cancer growth, we used the syngeneic murine Myc-Cap model [34] . Similar to DU145, PC3, and MC38OVA, in vitro treatment of Myc-Cap cells with JQ1 reduces PD-L1 expression and increases expression of the MHC Class I molecule H2K^q^. (Additional file 5: Figure S4). This model mimics the molecular properties of some human prostate cancers in that it exhibits AR amplification and overexpresses *c-myc* [34]. Previous work in our lab showed that anti-PD-1 treatment is ineffective in this model, and that an anti-CTLA-4 antibody of the isotype IgG2a has pre-clinical activity [35]. To model therapy for advanced prostate cancer, we treated robustly established tumors (≈ 450 mm^3^) with combination therapy using JQ1 and CTLA4 IgG2a. Consistent with our prior studies, anti-CTLA-4 resulted in significant tumor growth inhibition. BET bromodomain inhibition as a monotherapy was relatively ineffective; however combined treated showed a trend towards increased anti-tumor activity as compared to either treatment alone early in treatment (Fig. 5a-b) as well as a potential survival benefit (Fig. 5c-d). Animals treated with JQ1+ α-CTLA-4 had a 12.2% longer median survival than those treated with α-CTLA-4 alone (46 versus 41 days respectively), although that difference was not statistically significant. We next investigated immune correlates associated with combined treatment. α-CTLA-4 (IgG2a) increased CD8 infiltration (Fig. 5e), while decreasing the overall number of Tregs in the tumors (Fig. 5f). Clinically, an increased CD8:Treg ratio has been associated with improved outcome in a number of solid tumors [36, 37]; here, combined JQ1 + αCTLA-4 treatment showed a significantly increased CD8:Treg ratio as compared with α-CTLA-4 alone, and this increased ratio correlated with treatment effect (Fig. 5g). Intratumoral CD8 T cells from mice treated with the combination regimen showed a trend towards increased effector cytokine secretion (Fig. 5h-j). These in vivo data demonstrate a potential additive effect between BET Bromodomain and anti-CTLA-4, correlated primarily with an increased CD8:Treg ratio in the tumor. Further in vivo experiments with DU145 and PC3 xenografts confirmed the trends in PD-L1 and HLA-ABC expression seen in vitro. Ex vivo analysis of DU145 and PC3 tumors treated with JQ1 showed a trend towards decreased PD-L1 expression (Additional file 7: Figure S6A) and increased HLA-ABC expression (Additional file 7: Figure S6B), similar to the effects seen in treating DU145 and PC3 cells in vitro (Figs. 1-2).

## Discussion

Prostate cancer has remained relatively insensitive to immune checkpoint blockade [38], and this can be attributed to a number of factors, including a relatively low tumor mutation burden (TMB) [39], and a sparse infiltration of lymphocytes [40]. We found that inhibition of the BET Bromodomain BRD4 can reduce PD-L1 expression (Figs. 1b-e) and increase MHC Class I expression (Figs. 2a-d) on the surface of prostate tumor cells. These data are consistent with prior data using ovarian cancer cells [41]; here we extend those data significantly by demonstrating a contemporaneous increase in Class I MHC expression. Additionally, these conclusions were further borne out in xenograft models of DU145 and PC3, where JQ1 treatment decreased PD-L1 expression and increased Class I expression. (Additional file 7: FigureS6). In vivo CTL analyses demonstrated that these changes were immunologically relevant, as JQ1 pre-treatment resulted in increased susceptibility of tumor cells to antigen-specific CD8-mediated lysis (Fig. 3a-b). These trends were further corroborated in vivo of JQ1 treated MC38 tumors, in which PD-L1 expression was decreased, and there was a significant increase in OVA-specific CD8 infiltrate into the tumor (Fig. 3c-d).

On a broader level, our RNA-sequencing data using human prostate cancer cell lines showed that BET bromodomain inhibition alters the expression of a number of immune-related genes, and that JQ1 treatment further upregulates antigen presentation pathways initiated by IFN-γ treatment, while potentially decreasing signaling through certain growth factor pathways (Fig. 4). For example, *TAF9*, a subunit of the Transcription Factor IID (TFIID) required for initiation of transcription by RNA Polymerase II [32], is significantly upregulated by JQ1 (Fig. 4a, Additional file 5: Figure S4), but most significantly at the higher dose of JQ1. *TAF9* associates with CIITA, the MHC Class II transactivator [42], a complex responsible for upregulating Class I genes upon IFN-γ stimulation [43].

Interestingly, we found that JQ1 appears to differentially modulate expression of Class I MHC alleles. A similar phenomenon was previously shown to occur in the context of cytokine stimulation [44, 45]; in those studies differential expression was modulated by differential affinity of the NF-kB subunit RelA for the HLA-A and B promoter over HLA-C [45]. That pattern of expression is similar to what we found when bromodomain inhibition was added to IFN-γ treated cells, suggesting a potential common affinity based mechanism. It is also possible that JQ1 inhibits the transcription of one of the inhibitors of RelA/NF-kB activation, thus enhancing its ability to bind selectively to HLA-A and B to increase both mRNA and protein levels.

Immunologically, we further found that TRIM36, an E3 ubiquitin-protein ligase, was upregulated in a dose-dependent manner by BET Bromodomain inhibition combined with IFN-γ; increased TRIM36 expression has been correlated with inhibition of prostate cancer proliferation and cell-cycle progression through inhibition of the MAPK/ERK pathway [46]. TRIM36 is also involved in antigen processing [28]. Consistent with our data showing that PD-L1 is decreased with JQ1 treatment, other groups have shown that BRD4 binds directly to the PD-L1 promoter to mediate its transcription [41, 47, 48] such that inhibition of BRD4 would decrease the mRNA and protein levels of PD-L1. These genome-wide changes suggest that BET bromodomain inhibition can enhance the immunogenicity of prostate cancer while also acting on the tumor cells to inhibit their growth.

Taken together, these data demonstrate the activity of anti-CTLA-4 and BET bromodomain inhibition in a murine prostate cancer model. Previous work in a murine lymphoma model showed a similar decrease in PD-L1 upon treatment with JQ1 in vitro, and an additive effect of JQ1 and anti-PD-1 in prolonging survival in vivo [48]. Despite the differences between the lymphoma model and the Myc-Cap model, which is unresponsive to anti-PD-1 [35], the trends seen in PD-L1 decrease and improved tumor control are consistent. Our lab previously demonstrated the activity of anti-CTLA-4 in the Myc-Cap model [35] and showed that CTLA-4 immunotherapy significantly increased the production IFN-γ by CD8 and CD4 tumor-infiltrating T cells [35]. Mechanistically, the trend towards increased anti-tumor activity of combination treatment correlated with an increased CD8:Treg ratio and a trend toward increased intratumoral CD8 effector function. Although anti-CTLA-4 is not currently FDA-approved for the treatment of advanced prostate cancer, a randomized Phase III trial [49] showed a trend towards increased survival, suggesting that this agent may have some clinical activity. The data here suggest that activity could be augmented by BET bromodomain inhibition, leading to improved antitumor responses in the clinic.

## Materials and methods

### Cell culture

Human prostate cancer cell lines PC3 and DU145 were obtained from ATCC (Manassas, VA, USA) and grown in a monolayer under standard culture conditions, 5% CO2 in a 37 °C incubator, in RPMI 1640 (Corning, Corning, New York, USA) supplemented with 10% fetal bovine serum and 1% penicillin/streptomycin. Cell identity was confirmed by short tandem repeat (STR) typing. Cells were tested for mycoplasma by PCR. For flow cytometry studies, cells were plated at a density of ~ 5,000/cm^2^ into 6-well tissue culture plates or T-175 tissue culture flasks (for qPCR and RNA-Seq) and were allowed to adhere for 24 h. After 24 h, cells were treated with either 1 μM or 0.1 μM JQ1 (S7110, Selleckchem, Houston, TX, USA) and/or 100 units/mL of human interferon gamma (IFN-γ) (300–02, Pepro Tech, Rocky Hill, Connecticut, USA) for 48 h prior to harvest.

### Epigenetic drug screen

DU145 cells were plated as described above, and allowed to adhere for 24 h. After 24 h, cells were treated with either 100 units/mL of human interferon gamma (IFN-γ) (300–02, Pepro Tech, Rocky Hill, Connecticut, USA), or one of the following drugs at a concentration at approximately the published IC50 on the manufacturer’s product page: 0.01 μM GSK591 (S8111, Selleckchem), 0.1 μM GSK484 (17,488, Cayman Chemicals, Michigan, USA), 0.1 μM MS049 (18,348, Cayman Chemicals), 0.1 μM SGC707 (S7832, Selleckchem), 0.01 μM GSK343 (S7164, Selleckchem), 0.1 μM LLY-507 (S7575, Selleckchem), 0.1 μM A-196 (S7983, Selleckchem), 0.1 μM JQ1 (S7110, Selleckchem), 0.1 μM NVS-1 (5744, Tocris Biosciences, Bristol, UK), 0.1 μM LP-99 (17,661, Cayman Chemicals), 0.1 μM Entinostat (S1053, Selleckchem), or 0.1 μM 5-Azacytidine (S1782, Selleckchem) for 48 h prior to harvest. Cells were stained with Brilliant Violet 421™ (BV421) mouse anti-human PD-L1 1:50 (CD274, Clone 29E.2A3, BioLegend, San Diego, CA, USA) and eFluor™ 780 fixable viability dye 1:10000 (65,086,514, Invitrogen, Waltham, MA, USA) using the flow cytometry process detailed below.

### Flow Cytometry

PC3 and DU145 cells were stained with Brilliant Violet 421™ (BV421) mouse anti-human PD-L1 1:50 (CD274, Clone 29E.2A3, BioLegend, San Diego, CA, USA), PE mouse anti-human HLA-A,B,C 1:100 (Clone W6/32, BioLegend, San Diego, CA, USA), and eFluor™ 780 fixable viability dye 1:10000 (65,086,514, Invitrogen, Waltham, MA, USA). Staining was performed in PBS for 20 min at room temperature. Cells were analyzed using a BD FACSCelesta (Becton Dickinson, Franklin Lakes, New Jersey, USA) and FlowJo software (Tree Star, Ashland, Oregon, USA). The relative mean fluorescence intensity (MFI) was calculated as the MFI of each experimental sample/MFI of vehicle (DMSO) control.

### Quantitative real-time PCR

PC3 and DU145 cells were cultured and treated as indicated above. Total RNA was extracted using TRIzol reagent (15596–026, Invitrogen) followed by chloroform extraction as previously described [50]. cDNA was prepared from total RNA using the RNA to cDNA EcoDry Premix (Clontech, Mountain View, CA). qPCR detection of human CD274, HLA-A, HLA-B, and HLA-C were performed with TaqMan gene expression assays (Applied Biosystems, Foster City, CA, USA) according to protocol provided by manufacturer using TaqMan Universal Master Mix II no UNG (4,440,040, Applied Biosystems, Foster City, CA, USA) and analyzed on the ABI Viia 7 (Applied Biosystems). The ΔΔ CT method was used to quantify relative mRNA expression. Expression of each target gene was normalized to that of the reference gene 18S. qPCR wells were plated in triplicate and each assay was repeated at least 3 times.

### CTL assays

6–8 week old female OT-1 mice (003831, Jackson Laboratories, Farmington, CT, USA) were allowed to acclimate for 2 weeks after arrival to the facility. Animals were housed in specific pathogen-free facilities accredited by the American Association for the Accreditation of Laboratory Animal Care (AAALAC) with protocols approved by the Animal Care and Use Committee of the Columbia University School of Medicine (New York, NY). They were then sacrificed, and spleen and lymph nodes were excised. Following manual dissociation, cells were counted and resuspended at a concentration of 5 × 10^6^ cells/mL with 10 ng/mL of OVA SIINFEKL peptide (AS-60193-1, AnaSpec, Freemont, CA, USA), and plated at a density of about 2.5 × 10^6^ cells/cm^2^ in a tissue culture multi-well plate. After 48 h, cells were scraped, washed once with sterile PBS, and then isolated using Ficoll-Paque® PLUS (17–1440-03, GE Healthcare, Chicago, IL) according to manufacturer’s protocol in order to remove dead cells. These cells were then resuspended in media containing 10 ng/mL of murine IL-2 (212–12, PeproTech) and plated at 2.5 × 10^6^ cells/cm^2^ in a tissue culture multi-well plate to expand for an additional 24 h. OT1 CD8 cells were isolated using the mouse CD8a^+^ T Cell Isolation Kit (130–104-075, Miltenyi Biotec, Bergisch Gladbach, Germany) according to protocol provided by manufacturer. Lytic assays were performed using MC38-OVA cells plated in 6-well tissue culture plates at a starting density of ~ 5000/cm^2^ in DMEM (Corning, Corning, New York, USA) containing 10% fetal bovine serum and 1% penicillin/streptomycin under standard culture conditions, 5% CO2 in a 37 °C incubator. After 24 h, cells were treated with either 1 μM or 0.1 μM JQ1 (S7110, Selleckchem, Houston, TX, USA) and/or 20 ng/mL of murine interferon gamma (IFN-γ) (315–05, Pepro Tech, Rocky Hill, Connecticut, USA) for 48 h. Cells were then harvested by exposing cells to Trypsin-EDTA 0.05% (25,300,054, Gibco, Grand Island, New York, USA) for 1 min. The MC-38 OVA cells were then labeled with 20 μM PKH26 Red Fluorescent Cell Linker Kit (MINI26, Sigma-Aldrich, Saint Louis, MO, USA) according to protocol provided by manufacturer. The cells were washed thoroughly to remove residual PKH26 dye, and then pulsed with 10 ng/mL of OVA peptide (AnaSpec) for 4 h. Cells were then harvested by exposing cells to Trypsin as before, and then washed to remove residual peptide. 3 × 10^4^ target MC38-OVA cells were placed each well of a 6-well culture plate and allowed to adhere for 4 h, and OT1 CD8s activated as detailed above were then added to each well at the indicated Effector: Target ratios. After 12 h, cells were harvested and placed on ice to prevent additional killing. Cells were stained with eFluor™ 780 fixable viability dye 1:10000 (Invitrogen) to discern cell death on ice for 30 min, and were immediately analyzed using a BD FACSCelesta (Becton Dickinson) and FlowJo software (Tree Star). PKH26^+^Viability Dye^hi^ cells were considered lysed MC-38 OVA. Each treatment condition was plated in triplicate, and the assay was repeated twice.

### RNA-sequencing

RNA sequencing and analysis of treated DU145 and PC3 cells was performed by the JP Sulzberger Columbia Genome Center Core Facility. Total RNA was extracted from cells treated as above using Trizol Reagent (15596–026, Invitrogen). RNA purity was assessed ensure RIN was greater than 8. mRNA was enriched from total RNA (approx.. 150 ng/sample) using poly-A pull-down, and subsequently the Illumina TruSeq RNA prep kit (Illumina, San Diego, CA, USA) was used for library preparation. Libraries were sequenced using the Illumina HiSeq2500 (Illumina) with multiplexed samples in each line, which yields targeted number of single-end 100 bp reads for each sample, with an average of 30 million reads per sample. RTA (Illumina) was used for base calling, and bcl2fastq2 (version 2.17) was used for converting BCL to fastq format, coupled with adaptor trimming. Reads were mapped to the reference genome (Human: NCBI/build37.2; Mouse: UCSC/mm10) using STAR (2.5.2b) and featureCounts(v1.5.0-p3). Differential expression between groups were assessed using the R package DEseq, which uses a negative binomial distribution that models the number reads from RNA-seq experiments and test for differential expression. Three samples per condition per cell line were submitted for sequencing. Fragments per kilobase of transcript (FPKM) were used for downstream analysis including heatmaps and correlation matrix analysis. Spearman’s correlation was used to calculate pairwise comparisons for the immune genes and network analysis.

### Gene set enrichment analysis

Genes that were significantly differentially expressed (*P* < 0.05) between the DMSO treated group and the IFN-γ treated group were compared to the list of genes that were significantly differentially expressed between the IFN-γ treated group and IFN-γ + 1 μM JQ1 treated group. Genes that appeared on both lists were then separated into those that were upregulated or downregulated based on positive or negative fold-change in the DEseq analysis. The magnitude of the fold-change was used to discern genes that were the most upregulated or downregulated. The list of the 20 most upregulated and downregulated genes were input into EnrichR [30, 31] to assess changes in Reactome pathways. The significance of pathways were ranked using the combined score, which is a combination of the *p*-value and z-score calculated as previously described [30].

### In-vivo tumor models

Myc-Cap cells were obtained from ATCC (Manassas, VA, USA) and grown in a monolayer under standard culture conditions, 5% CO2 in a 37 °C incubator, in DMEM 1640 (Corning, Corning, New York, USA) supplemented with 10% fetal bovine serum and 1% penicillin/streptomycin. 1 × 10^6^ Myc-Cap cells were implanted subcutaneously into 8–10 week old male FVB/NJ mice (001800, Jackson Laboratories, Farmington, CT, USA) in the right flank as previously described [51]. Animals were housed in specific pathogen-free facilities accredited by the American Association for the Accreditation of Laboratory Animal Care (AAALAC) with protocols approved by the Animal Care and Use Committee of the Columbia University School of Medicine (New York, NY). Tumor dimensions were measured with an electronic caliper every 2–3 days as indicated. Overall tumor volumes were calculated as 0.5 × longest diameter × shortest diameter^2^. Mice were randomized into treatment groups as indicated on day 26 when tumors reached approximately 450 mm^3^, receiving either Vehicle (5% DMSO in 10% 2-hydroxypropyl-β-cyclodextrin) + IgG2a isotype control, 50 mg/kg JQ1 via intraperitoneal (IP) injection daily, 10 mg/kg aCTLA-4 (IgG2a) IP every other day for 3 doses, or both. Mice were euthanized once tumors exceeded 2 cm in either dimension, or when tumors became ulcerated or mice showed signs of dehydration/illness. Anti–CTLA-4 and the corresponding IgG2a isotype control was a generous gift from Dr. Alan Korman and Dr. Mark Selby, provided under an MTA with Bristol-Myers Squibb (Redwood City, CA).

MC38OVA cells were a generous gift from Dr. Mark Smyth (Melbourne, Australia) and grown in a monolayer under standard culture conditions, 5% CO2 in a 37 °C incubator, in DMEM 1640 (Corning, Corning, New York, USA) supplemented with 10% fetal bovine serum and 1% penicillin/streptomycin. 5 × 10^5^ MC38OVA cells were implanted subcutaneously into 8–10 week old male C57/BL6 mice (000664, Jackson Laboratories, Farmington, CT, USA) in the right flank as previously described [51]. Animals were housed in specific pathogen-free facilities accredited by the American Association for the Accreditation of Laboratory Animal Care (AAALAC) with protocols approved by the Animal Care and Use Committee of the Columbia University School of Medicine (New York, NY). Tumor dimensions were measured with an electronic caliper every 2–3 days as indicated. Overall tumor volumes were calculated as 0.5 × longest diameter × shortest diameter^2^. Mice were randomized into treatment groups as indicated on day 11 when tumors reached approximately 150 mm^3^, receiving either Vehicle (5% DMSO in 10% 2-hydroxypropyl-β-cyclodextrin) or 50 mg/kg JQ1 via intraperitoneal (IP) injection daily. Mice were sacrificed on d17 for ex vivo flow cytometry analysis of tumor PD-L1 (Clone 10F.9G2, BioLegend) expression as well as OVA-specific CD8 TIL using OVA Tetramer staining (MBL International, Woburn, MA, USA).

DU145 and PC3 cells were obtained from ATCC (Manassas, VA, USA) and grown in a monolayer under standard culture conditions, 5% CO2 in a 37 °C incubator, in RPMI 1640 (Corning, Corning, New York, USA) supplemented with 10% fetal bovine serum and 1% penicillin/streptomycin. 3 × 10^6^ DU145 or PC3 cells were implanted subcutaneously into 8–10 week old male J:Nu mice (007850, Jackson Laboratories, Farmington, CT, USA) in the right flank as previously described [51]. Animals were housed in specific pathogen-free facilities accredited by the American Association for the Accreditation of Laboratory Animal Care (AAALAC) with protocols approved by the Animal Care and Use Committee of the Columbia University School of Medicine (New York, NY). Tumor dimensions were measured with an electronic caliper every 2–3 days as indicated. Overall tumor volumes were calculated as 0.5 × longest diameter × shortest diameter^2^. Mice were randomized into treatment groups as indicated on day 14 when tumors reached approximately 150 mm^3^, receiving either Vehicle (5% DMSO in 10% 2-hydroxypropyl-β-cyclodextrin) or 50 mg/kg JQ1 via intraperitoneal (IP) injection daily. Mice were sacrificed on d20 for ex vivo flow cytometry analysis of tumor PD-L1 and HLA-ABC expression using the same antibodies as used for in vitro assays.

### Flow Cytometry analysis of tumor infiltrating lymphocytes (TIL)

Single cell suspensions were prepared from Myc-Cap tumors via mechanical dissociation using Mouse Tumor Dissociation Kit (Miltenyi) on program m_TDK_1 using a gentleMACS Octo Dissociator (Miltenyi) according to manufacturer protocol. Cells were then stained with the following flourochrome-conjugated antibodies: Panel 1 - CD45 BV421 (Clone 30-F11, BioLegend), CD8 BV510 (Clone 53–6.7, BioLegend), CD4 BV650 (Clone RM4–5, BioLegend), CTLA-4 PE-TexasRed (Clone UC10-4B9, BioLegend), PD-1 PE/Cy7 (Clone 29F.1A12, BioLegend), TCRb AF700 (Clone H57–597, BioLegend); Panel 2 – CTLA-4 BV421 (Clone UC10-4B9, BioLegend), PD-1 BV605 (Clone 29F.1A12, BioLegend), CD4 BV650 (Clone RM4–5, BioLegend), CD8 AF700 (Clone 53–6.7, BioLegend). Intracellular stains were as follows: Panel 1 – CTLA-4 PE-TexasRed (Clone UC10-4B9, BioLegend), FoxP3 FITC (Clone FJK-16 s, Invitrogen); Panel 2 – CTLA-4 BV421 (Clone UC10-4B9, BioLegend), TNFα FITC (Clone MP6-XT22, BD), Granzyme B APC (Clone BG11, Invitrogen). For intracellular staining, cells were fixed and permeabilized using fixation/permeabilization concentrate and diluent (eBiosciences, San Diego, CA) at room temperature for 45 min. For intracellular cytokine staining, cells were stimulated with PMA (50 ng/ml)/ionomycin (500 ng/ml) for 4 h in the presence of protein transport inhibitor cocktail (eBiosciences, San Diego, CA). Cells were analyzed using a BD FACSCelesta (Becton Dickinson, Franklin Lakes, New Jersey, USA) and FlowJo software (Tree Star, Ashland, Oregon, USA).

### Statistical methods

GraphPad Prism 6 and R (3.4.3, CRAN) were used for statistical analysis. Quantitative data are shown as mean ± Standard Error of the Mean (SEM) unless otherwise stated. For all comparisons, groups were compared to the DMSO (vehicle) treated group unless otherwise indicated. Multiplicity adjusted *P*-values from one-way ANOVA analysis was used to determine significance, with a *p* < 0.05 considered significant. Family-wise significance was set to 0.05 (95% confidence interval).

## Supplementary information

**Additional file 1: Table S1.** List of inhibitors used to target epigenetic modifiers. The major classes of epigenetic modulators are abbreviated MT = methyltransferase; PAD = protein arginine deiminase; BRD = bromodomain; HDAC = Histone deacetylase.

**Additional file 2: Figure S1.** Effects of BET Bromodomain Inhibition on proliferation of DU145 and PC3 cells. **A.** % Cell proliferation of DU145 treated with JQ1 at indicated concentrations as measured through MTT assay. *N* = 2/iteration, repeated × 3. All comparisons made to DMSO treatment. **B.** % Cell proliferation of PC3 treated with JQ1 at indicated concentrations as measured through MTT assay. *N* = 2/iteration, repeated × 3. All comparisons made to DMSO treatment.

**Additional file 3: Figure S2.** BD2 Inhibition Downregulates PD-L1 Expression in Prostate Cancer. **A.** Histograms of PD-L1 expression in PC3 cells treated with RVX208 and/or IFNγ with MFI as indicated, gated on live cells. *N* = 1/iteration, repeated × 1. **B.** Summary flow cytometry data for A. *N* = 1/iteration, repeated × 1.

**Additional file 4: Figure S3.** BET Bromodomain Inhibition Downregulates PD-L1 and Augments MHC I Expression in MC38OVA. **A.** Representative histograms of PD-L1 expression in MC38OVA cells treated with JQ1 and/or IFNγ gated on live cells. **B.** Summary flow cytometry data for A. *N* = 1 sample / iteration, repeated × 3. **C.** Representative histograms of H2K^b^D^b^ expression in MC38OVA cells treated with JQ1 and/or IFNγ gated on live cells. **D.** Summary flow cytometry data for C. *N* = 1 sample / iteration, repeated × 3. * *p* < 0.05, **p < 0.01, *** *p* < 0.001, ****p < 0.0001. Error bars = standard deviation.

**Additional file 5: Figure S4.** BET Bromodomain Inhibition Downregulates PD-L1 and Augments MHC I Expression in Myc-Cap. **A.** Summary flow cytometry data of PD-L1 expression in Myc-Cap cells treated with JQ1 and/or IFNγ, normalized to DMSO. *N* = 2 samples / iteration, repeated × 3. **B.** Summary flow cytometry data of H2K^q^ expression in Myc-Cap cells treated with JQ1 and/or IFNγ, normalized to DMSO. *N* = 2 samples / iteration, repeated × 3.

**Additional file 6: Figure S5.** Global effects of BET Bromodomain Inhibition on Prostate Cancer. **A.** Differentially expressed genes between DU145 cells treated as indicated. **B.** Top 20 upregulated and downregulated genes between the 1uM JQ1 + IFNγ vs IFNγ treated groups in PC3 cells. *p* < 0.05 for all significantly differentially expressed genes. *N* = 3/group, repeated × 1.

**Additional file 7: Figure S6.** BET Bromodomain Inhibition Downregulates PD-L1 and HLA-ABC in DU145 and PC3 xenograft tumors. **C.** Summary of flow cytometry data for PD-L1 staining in ex-vivo DU145 and PC3 tumors, gated on Live CD45^−^ cells. *N* = 6 mice / iteration, repeated × 2. **D.** Summary of flow cytometry data for HLA-ABC staining in ex-vivo MC38OVA tumors gated on Live CD45^−^ cells. *N* = 6 mice / iteration, repeated × 2.

## Data Availability

The RNA-Seq datasets generated during and/or analyzed during the current study are available in the NCBI Gene Expression Omnibus (GEO) repository, study number GSE126779.
