# Supplementary material for: Immunogenicity of prostate cancer is augmented by BET bromodomain inhibition
Source: J Immunother Cancer. 2019 Oct 25;7:277. doi: 10.1186/s40425-019-0758-y (PMC6814994; doi:10.1186/s40425-019-0758-y)
Supplement: Supplementary file 1 — Additional file 1: Table S1. List of inhibitors used to target epigenetic modifiers. The major classes of epigenetic modulators are abbreviated MT = methyltransferase; PAD = protein arginine deiminase; BRD = bromodomain; HDAC = Histone deacetylase. [file 40425_2019_758_MOESM1_ESM.pptx]

## Slide 1
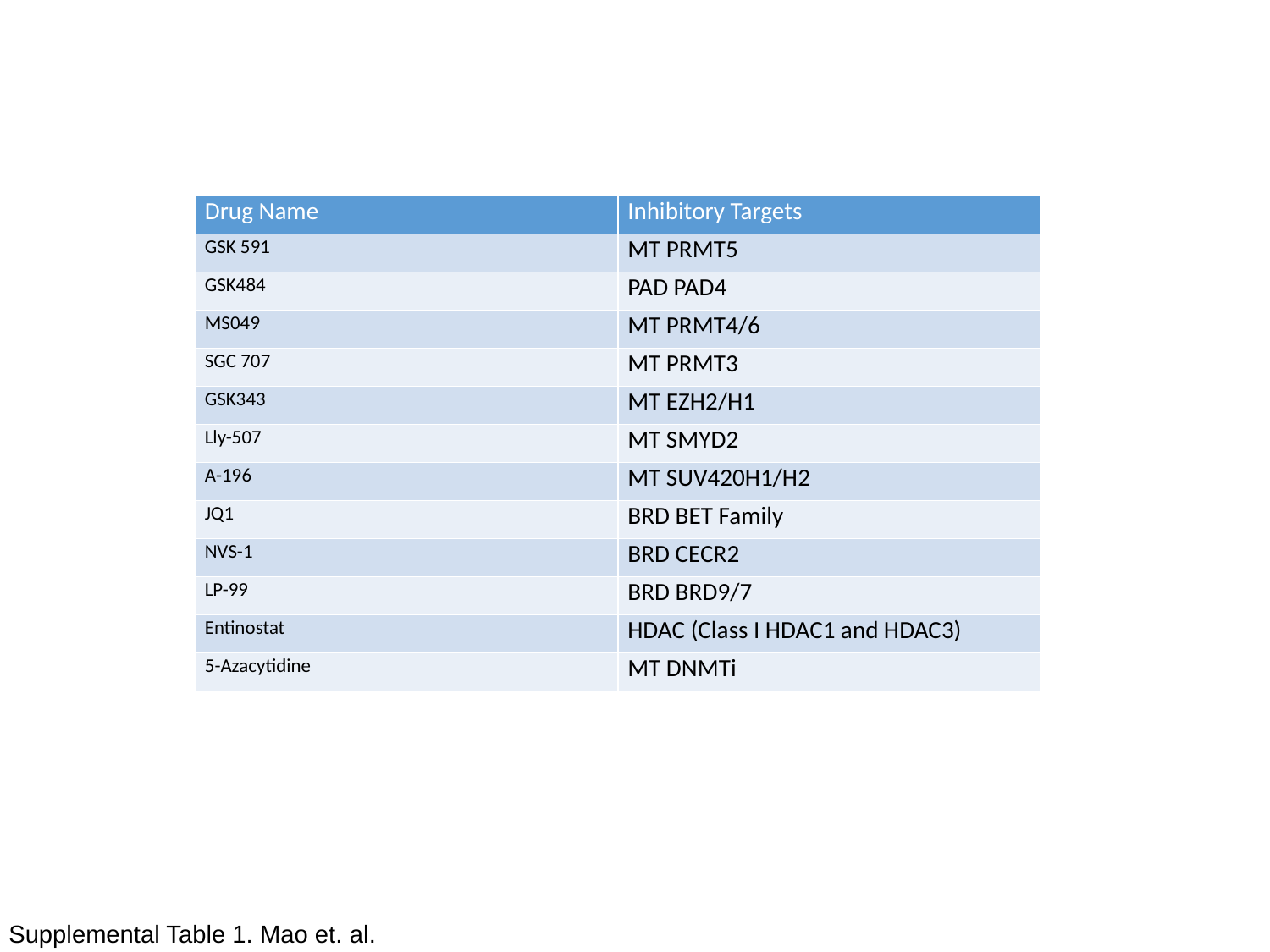

| Drug Name | Inhibitory Targets |
| --- | --- |
| GSK 591 | MT PRMT5 |
| GSK484 | PAD PAD4 |
| MS049 | MT PRMT4/6 |
| SGC 707 | MT PRMT3 |
| GSK343 | MT EZH2/H1 |
| Lly-507 | MT SMYD2 |
| A-196 | MT SUV420H1/H2 |
| JQ1 | BRD BET Family |
| NVS-1 | BRD CECR2 |
| LP-99 | BRD BRD9/7 |
| Entinostat | HDAC (Class I HDAC1 and HDAC3) |
| 5-Azacytidine | MT DNMTi |
Supplemental Table 1. Mao et. al.
