# Supplementary figures and images for: Immunogenicity of prostate cancer is augmented by BET bromodomain inhibition
Source: J Immunother Cancer. 2019 Oct 25;7:277. doi: 10.1186/s40425-019-0758-y (PMC6814994; doi:10.1186/s40425-019-0758-y)

## Slide 1
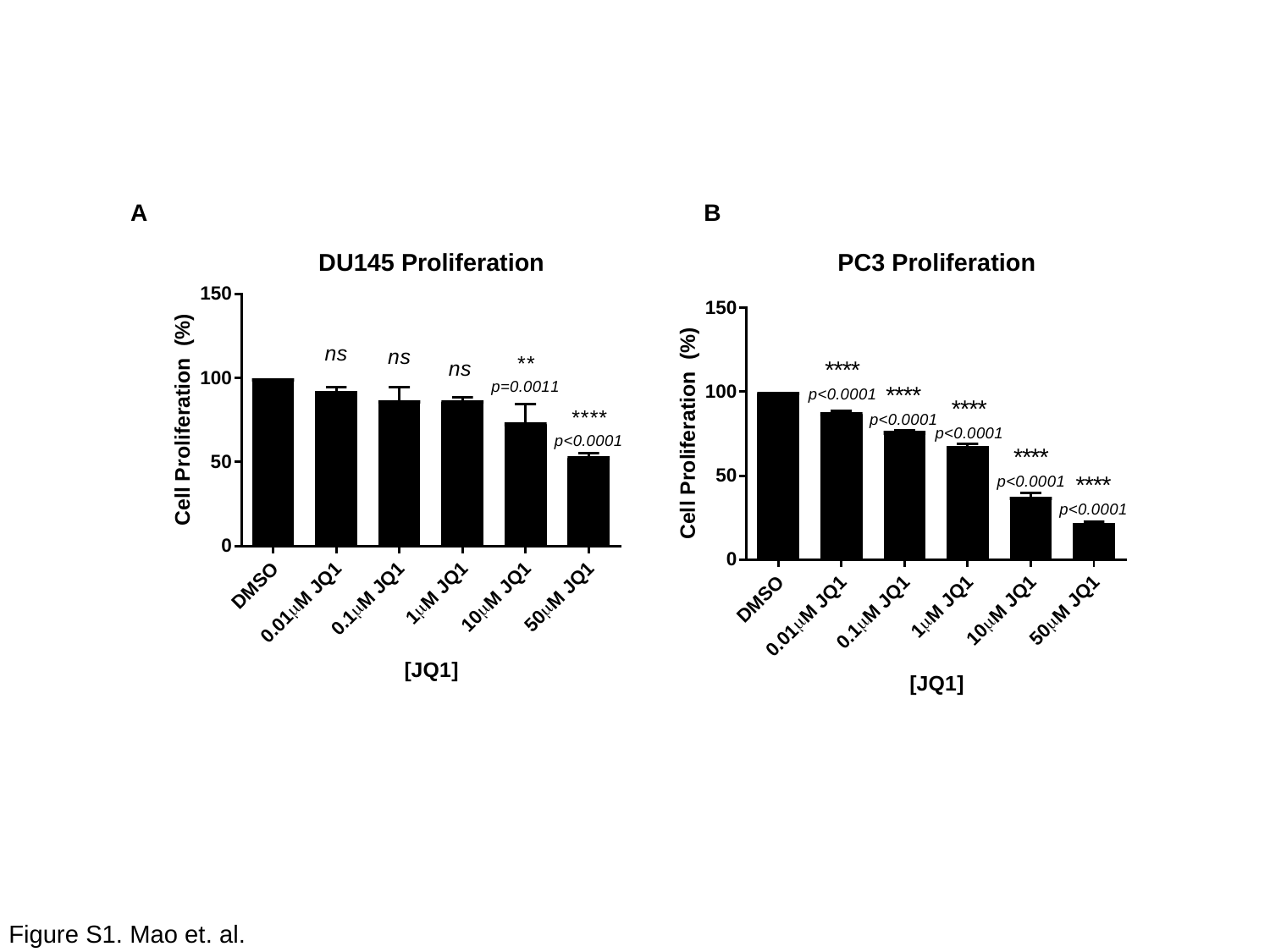

B
A
Figure S1. Mao et. al.

Supplement: Supplementary file 2 — Additional file 2: Figure S1. Effects of BET Bromodomain Inhibition on proliferation of DU145 and PC3 cells. A. % Cell proliferation of DU145 treated with JQ1 at indicated concentrations as measured through MTT assay. N = 2/iteration, repeated × 3. All comparisons made to DMSO treatment. B. % Cell proliferation of PC3 treated with JQ1 at indicated concentrations as measured through MTT assay. N = 2/iteration, repeated × 3. All comparisons made to DMSO treatment. [file 40425_2019_758_MOESM2_ESM.pptx]

## Slide 1
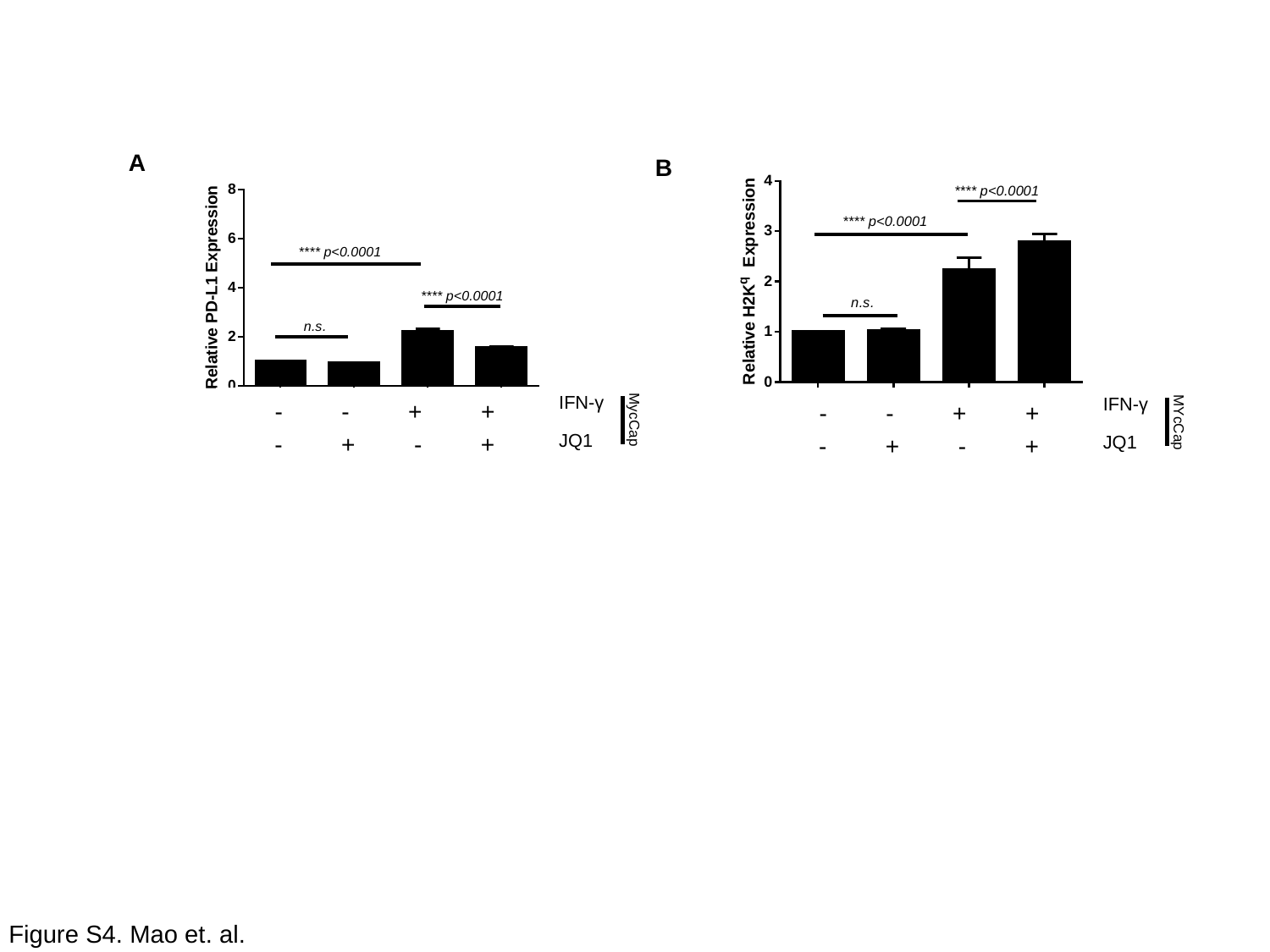

A
B
IFN-γ
JQ1
IFN-γ
JQ1
- - + +
- + - +
- - + +
- + - +
MycCap
MYcCap
Figure S4. Mao et. al.

Supplement: Supplementary file 5 — Additional file 5: Figure S4. BET Bromodomain Inhibition Downregulates PD-L1 and Augments MHC I Expression in Myc-Cap. A. Summary flow cytometry data of PD-L1 expression in Myc-Cap cells treated with JQ1 and/or IFNγ, normalized to DMSO. N = 2 samples / iteration, repeated × 3. B. Summary flow cytometry data of H2Kq expression in Myc-Cap cells treated with JQ1 and/or IFNγ, normalized to DMSO. N = 2 samples / iteration, repeated × 3. [file 40425_2019_758_MOESM5_ESM.pptx]

## Slide 1
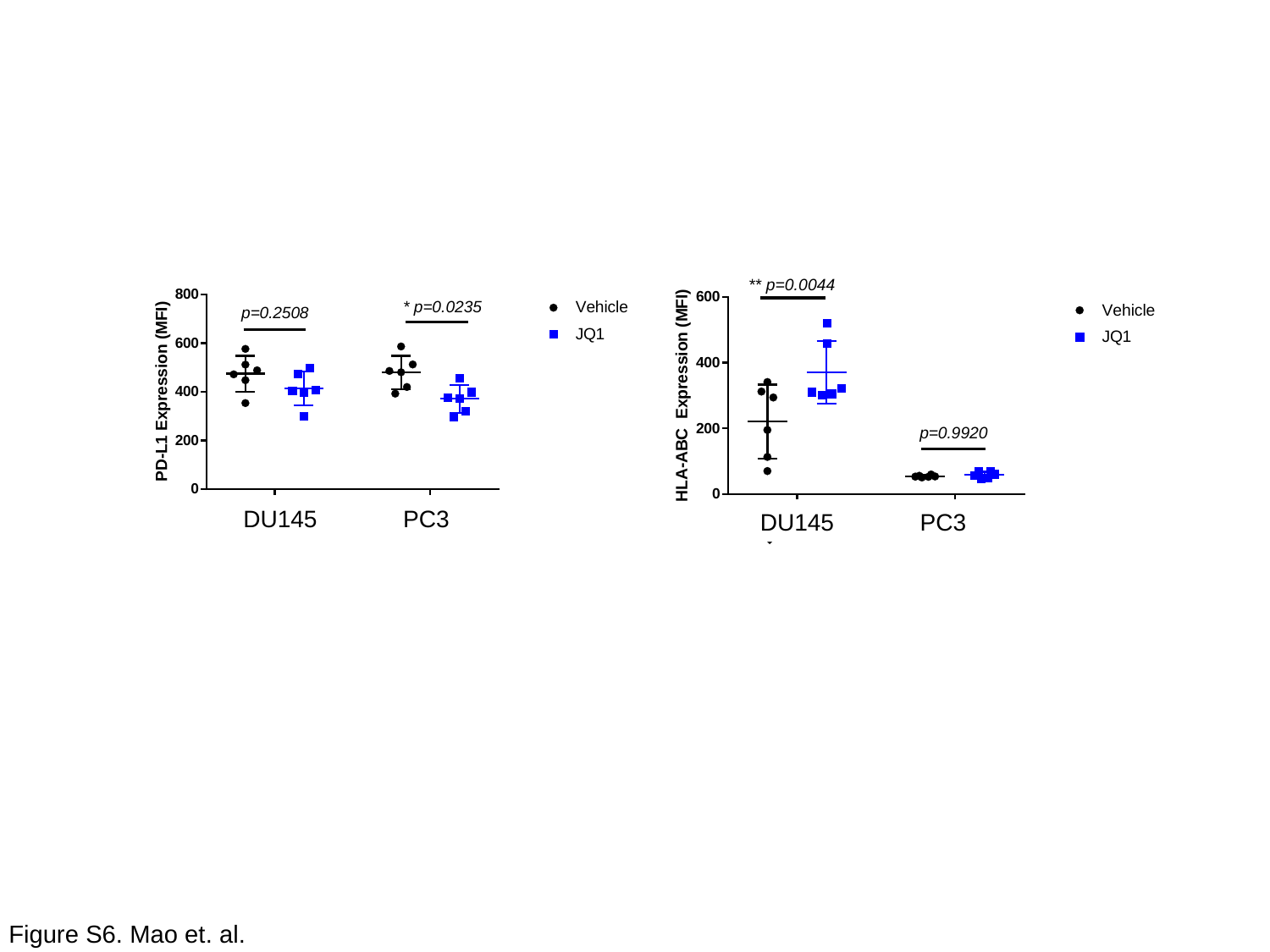

DU145	 PC3
DU145	 PC3
Figure S6. Mao et. al.

Supplement: Supplementary file 7 — Additional file 7: Figure S6. BET Bromodomain Inhibition Downregulates PD-L1 and HLA-ABC in DU145 and PC3 xenograft tumors. C. Summary of flow cytometry data for PD-L1 staining in ex-vivo DU145 and PC3 tumors, gated on Live CD45− cells. N = 6 mice / iteration, repeated × 2. D. Summary of flow cytometry data for HLA-ABC staining in ex-vivo MC38OVA tumors gated on Live CD45− cells. N = 6 mice / iteration, repeated × 2. [file 40425_2019_758_MOESM7_ESM.pptx]
