# Supplementary material for: Immunogenicity of prostate cancer is augmented by BET bromodomain inhibition
Source: J Immunother Cancer. 2019 Oct 25;7:277. doi: 10.1186/s40425-019-0758-y (PMC6814994; doi:10.1186/s40425-019-0758-y)
Supplement: Supplementary file 3 — Additional file 3: Figure S2. BD2 Inhibition Downregulates PD-L1 Expression in Prostate Cancer. A. Histograms of PD-L1 expression in PC3 cells treated with RVX208 and/or IFNγ with MFI as indicated, gated on live cells. N = 1/iteration, repeated × 1. B. Summary flow cytometry data for A. N = 1/iteration, repeated × 1. [file 40425_2019_758_MOESM3_ESM.pptx]

## Slide 1
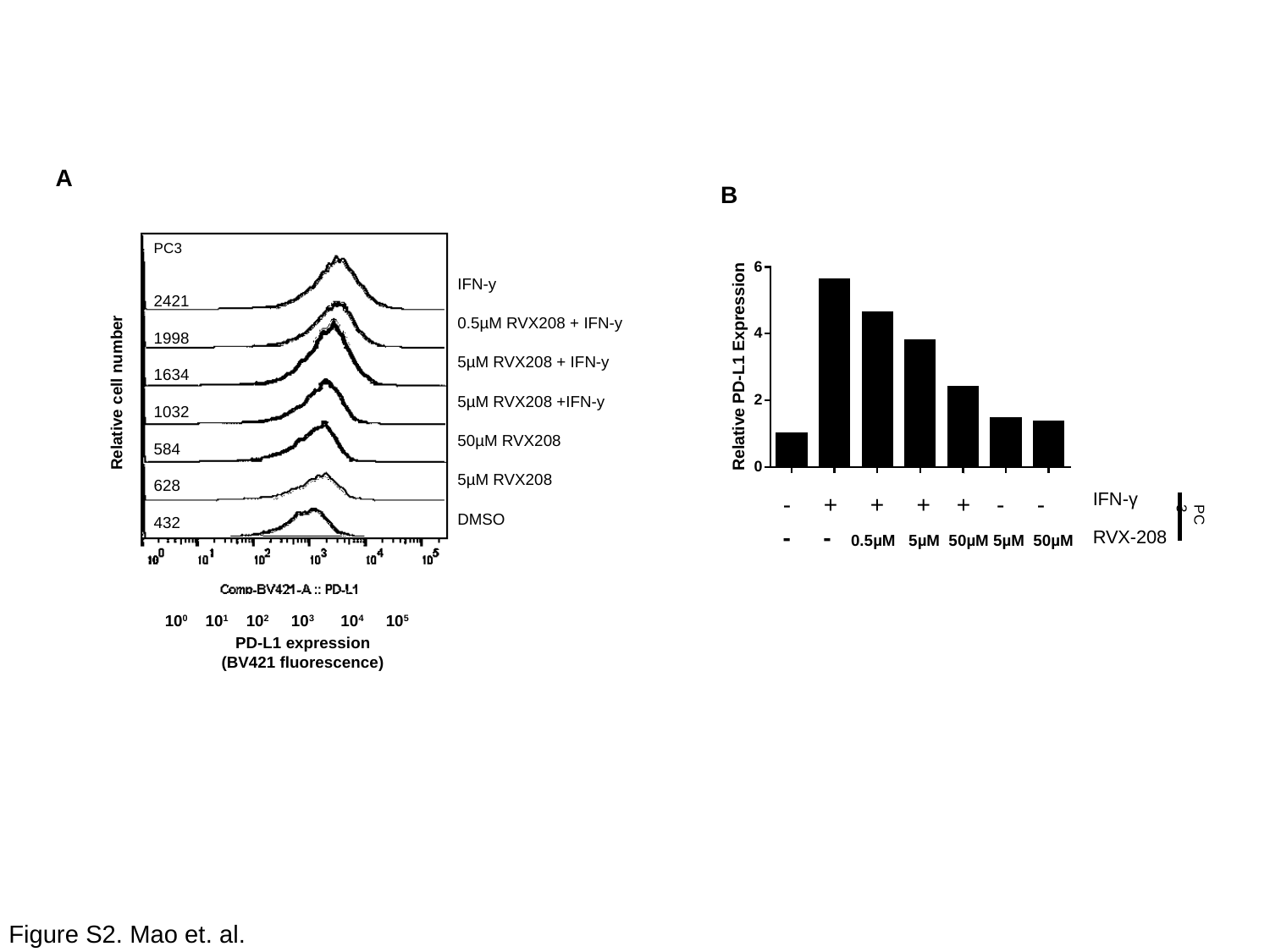

A
B
PC3
IFN-y
0.5µM RVX208 + IFN-y
5µM RVX208 + IFN-y
5µM RVX208 +IFN-y
50µM RVX208
5µM RVX208
DMSO
2421
1998
1634
1032
584
628
432
Relative cell number
IFN-γ
RVX-208
- + + + + - -
- - 0.5µM 5µM 50µM 5µM 50µM
PC3
100 101 102 103 104 105
PD-L1 expression
(BV421 fluorescence)
Figure S2. Mao et. al.
