# Supplementary material for: Immunogenicity of prostate cancer is augmented by BET bromodomain inhibition
Source: J Immunother Cancer. 2019 Oct 25;7:277. doi: 10.1186/s40425-019-0758-y (PMC6814994; doi:10.1186/s40425-019-0758-y)
Supplement: Supplementary file 4 — Additional file 4: Figure S3. BET Bromodomain Inhibition Downregulates PD-L1 and Augments MHC I Expression in MC38OVA. A. Representative histograms of PD-L1 expression in MC38OVA cells treated with JQ1 and/or IFNγ gated on live cells. B. Summary flow cytometry data for A. N = 1 sample / iteration, repeated × 3. C. Representative histograms of H2KbDb expression in MC38OVA cells treated with JQ1 and/or IFNγ gated on live cells. D. Summary flow cytometry data for C. N = 1 sample / iteration, repeated × 3. * p < 0.05, **p < 0.01, *** p < 0.001, ****p < 0.0001. Error bars = standard deviation. [file 40425_2019_758_MOESM4_ESM.pptx]

## Slide 1
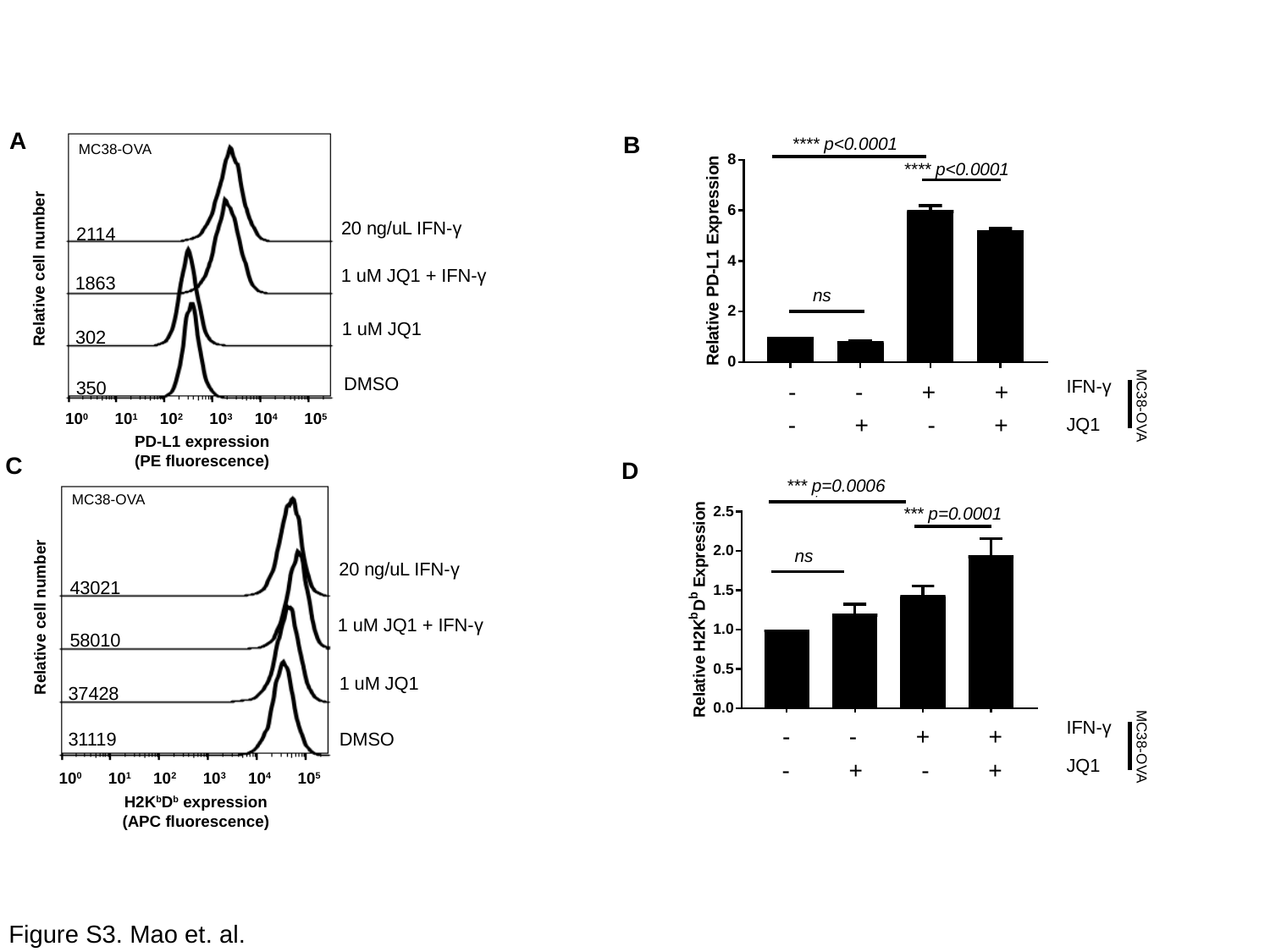

A
B
**** p<0.0001
MC38-OVA
**** p<0.0001
20 ng/uL IFN-γ
1 uM JQ1 + IFN-γ
1 uM JQ1
DMSO
2114
1863
302
350
Relative cell number
ns
IFN-γ
JQ1
- - + +
- + - +
MC38-OVA
100 101 102 103 104 105
PD-L1 expression
(PE fluorescence)
C
D
*** p=0.0006
MC38-OVA
*** p=0.0001
ns
20 ng/uL IFN-γ
1 uM JQ1 + IFN-γ
1 uM JQ1
DMSO
43021
58010
37428
31119
Relative cell number
IFN-γ
JQ1
- - + +
- + - +
MC38-OVA
100 101 102 103 104 105
H2KbDb expression
(APC fluorescence)
Figure S3. Mao et. al.
