# Supplementary material for: Immunogenicity of prostate cancer is augmented by BET bromodomain inhibition
Source: J Immunother Cancer. 2019 Oct 25;7:277. doi: 10.1186/s40425-019-0758-y (PMC6814994; doi:10.1186/s40425-019-0758-y)
Supplement: Supplementary file 6 — Additional file 6: Figure S5. Global effects of BET Bromodomain Inhibition on Prostate Cancer. A. Differentially expressed genes between DU145 cells treated as indicated. B. Top 20 upregulated and downregulated genes between the 1uM JQ1 + IFNγ vs IFNγ treated groups in PC3 cells. p < 0.05 for all significantly differentially expressed genes. N = 3/group, repeated × 1. [file 40425_2019_758_MOESM6_ESM.pptx]

## Slide 1
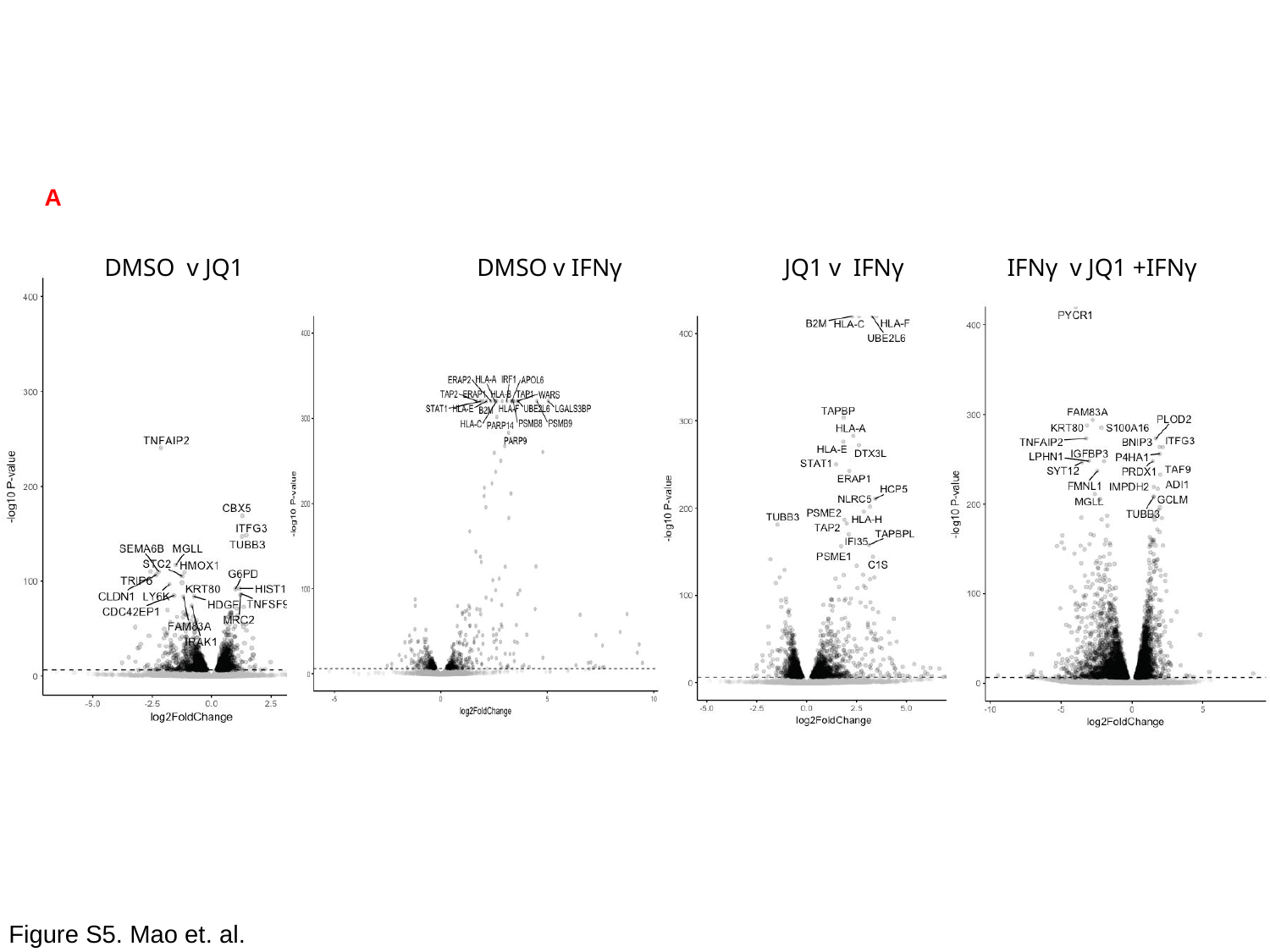

A
DMSO v JQ1
DMSO v IFNγ
JQ1 v IFNγ
IFNγ v JQ1 +IFNγ
Figure S5. Mao et. al.

## Slide 2
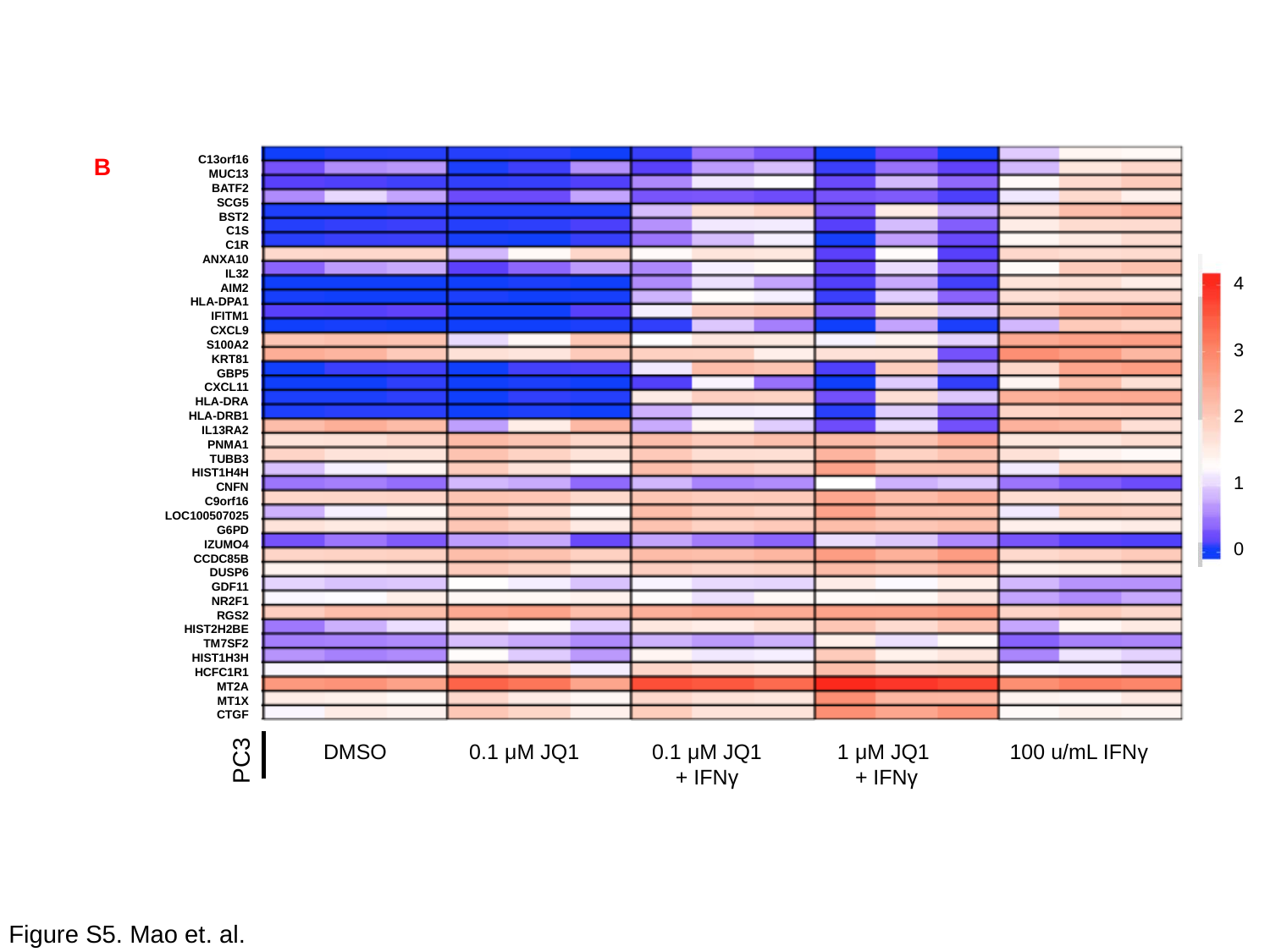

B
C13orf16
MUC13
BATF2
SCG5
BST2
C1S
C1R
ANXA10
IL32
AIM2
HLA-DPA1
IFITM1
CXCL9
S100A2
KRT81
GBP5
CXCL11
HLA-DRA
HLA-DRB1
IL13RA2
PNMA1
TUBB3
HIST1H4H
CNFN
C9orf16
LOC100507025
G6PD
IZUMO4
CCDC85B
DUSP6
GDF11
NR2F1
RGS2
HIST2H2BE
TM7SF2
HIST1H3H
HCFC1R1
MT2A
MT1X
CTGF
4
3
2
1
0
PC3
DMSO
0.1 μM JQ1
0.1 μM JQ1
+ IFNγ
1 μM JQ1
+ IFNγ
100 u/mL IFNγ
Figure S5. Mao et. al.
